# Current drawbacks and future perspectives in the diagnosis and treatment of male factor infertility, with a focus on FSH treatment: an expert opinion

**DOI:** 10.1007/s40618-024-02524-x

**Published:** 2025-01-13

**Authors:** D. Santi, G. Corona, A. Salonia, A. Ferlin

**Affiliations:** 1 Unit of Endocrinology, Department of Biomedical, Metabolic and Neural Sciences, University of Modena and Reggio Emilia, Modena, Italy; 2 Endocrinology Unit, Azienda AUSL, Bologna, Italy; 3 Division of Experimental Oncology/Unit of Urology, URI, IRCCS Ospedale San Raffaele, Milan, Italy; 4 Vita-Salute San Raffaele University, Milan, Italy; 5 Department of Medicine, University of Padova, Padua, Italy; 6 Unit of Andrology and Reproductive Medicine, Department of Systems Medicine, University Hospital of Padova, Via Giustiniani 2, 35121 Padua, Italy

**Keywords:** Male factor infertility, FSH supplementation, ART, Andrological workup

## Abstract

**Purpose:**

Infertility is defined as the inability to conceive after 1 year of unprotected intercourse, affecting approximately 15–20% of couples in Western countries. It is a shared problem within the couple; when the main issue lies with one of the partners, it is preferable to refer to “male factor” or “female factor” infertility rather than simply male or female infertility. Despite male factor infertility accounting for half of all couple infertility cases, the clinical approach to the male partner is not uniformly standardized across international guidelines.

**Methods:**

To provide an expert overview, we have comprehensively reviewed and critically analyzed the most up-to-date literature on this sensitive topic, leading to the development of a proposal for tailored assessment of the diagnostic-therapeutic pathway and preventive strategies. The diagnostic approach also considers that infertile men are objectively less healthy than their fertile counterparts of the same age and ethnicity.

**Results:**

This article discusses the diagnostic flow, the classification of male factor infertility, the definition of idiopathic infertility, the involvement of general health, and treatment recommendations, emphasizing follicle-stimulating hormone treatment in selected groups of patients.

**Conclusion:**

We provide expert opinion on current drawbacks and future perspectives in this field, with practical advice for the clinical practice of general practitioners and expert in reproductive medicine.

## Introduction

Infertility is defined as the inability to conceive after 1 year of unprotected intercourse, affecting approximately 15–20% of couples in Western countries [1]. It is important to clarify that male and female factors are equally represented, whether alone or in combination. Infertility should not be viewed a matter solely related to the woman or the man; it is always a problem involving the couple. Consequently, infertility should always be referred to as “couple infertility”. When the main problem lies with one partner, it is preferable to use the terms “male factor” or “female factor” infertility, rather than simply “male infertility” or “female infertility”. This approach means that the diagnostic and therapeutic process should be performed in parallel for both partners. Therefore, gynaecologists and andrologists should jointly proceed in the management work up of the couple [2]. Interestingly, the recent Italian guidelines from the Ministry of Health dealing with the management of infertility and Assisted Reproductive Techniques (ART) recommend that both partners be referred to reproductive specialists for their specific competence (i.e., the gynaecologists and the andrologists, respectively). Furthermore, the definition of male factor infertility could be determined only by andrologists [3].

Nevertheless, a common malpractice in real-life settings is to focus attention on only one partner, typically the female. However, the fertility potential of each partner and/or specific conditions affecting even only one member of the couple can profoundly influence the clinical and treatment approach. For example, mild reduction of sperm number and/or quality might be compatible with natural fertility if the female is at her top fertility capability (e.g. young age, absence of infertility causes, and risk factors). However, the same condition might be a cofactor of couple infertility when the fertility potential of the partner is reduced (e.g., older age, presence of infertility causes, and risk factors). Therefore, it is important to stress that the results of diagnostic tests should be interpreted within the context of the specific couple rather than as independent predictors of (in)fertility outcome.

Consequently, the clinical approach to couple infertility should be tailored considering the two individuals and specific aspects of that particular couple. As in other areas of clinical medicine, only a correct and extensive work up can direct further therapy and influence prognosis.

Although male factor infertility accounts for half of the cases of couple infertility, the clinical approach to the male partner is not uniformly standardized across international guidelines. The diagnostic approach is frequently limited to semen analysis, and any treatment for the male partner might be overlooked entirely, with the couple being directly referred to ART. Indeed, the aim of investigation is to achieve a diagnosis based on etiologic and pathophysiologic mechanisms, which will both guide the appropriate treatment and validate prognostic values for the couple’s fertility outcome.

In this context, male factor infertility can be caused by a variety of aetiologies and pathophysiologic mechanisms, many risk factors might be involved, and it might also reflect the general health of a man [4–6]. Furthermore, regardless of their ability to conceive naturally or by ART, identifying individuals with signs of spermatogenic and endocrine testicular impairment is clinically crucial for their follow-up and prognosis.

The present document summarizes the current literature and provides an expert opinion on the management of male factor infertility, highlighting current drawbacks and future perspectives to improve patients’ and couple’s care.

## Methods

Despite existing national and international guidelines providing physicians with guidance for addressing couple’s infertility, male factor infertility remains a significant challenge for clinicians in their everyday practice [7, 8]. These guidelines, developed by the various expert panels, aim to prioritize clinically relevant care decisions. The strength of each recommendation lies in a careful balance between different diagnostic and therapeutic options, the quality of available scientific evidence (i.e., mostly condensed in terms of systematic reviews and meta-analyses), and the nature and heterogeneity of patient values and preferences. This detailed decision-making process informs the strength rating of each guideline statement.

As clinicians deeply involved in the daily management of couples with male factor infertility and as translational researchers variously responsible for some of these guidelines, we have identified a potential gap. Specifically, while the guidelines are scientifically rigorous, they may lack flexibility for effective customization to the individual man, sometimes translating at a more “local” level what the suggestions in the guidelines propose in a more "absolutist" way [9].

For the specific purposes of this manuscript, we did not conduct a systematic review and meta-analysis of all published retrospective or prospective studies dealing with the management of the male factor infertility. Instead, we comprehensively examined and critically reworked all the most up-to-date literature on this sensitive topic. Using a scientifically rigorous yet accessible approach, we developed a proposal for a tailored evaluation of both diagnostic and therapeutic pathways, as well as preventive strategies. This proposal is also based literature evidence pointing out that infertile men are objectively less healthy than their fertile counterparts of the same ethnicity and age.

## Semen analysis is the starting point, not the final diagnosis

Semen analysis is undoubtedly a crucial component in investigating the male partner of an infertile couple, but its results should not be used alone to pose the diagnosis. Caution should be used when drawing conclusions about the fertility status of the patient and the couple. Indeed, semen analysis should be interpreted together with other clinical and diagnostic procedures to assess the fertility potential and the health of the genital tract. In other words, semen analysis is not an absolute indicator of the (in)fertility of the patient and the couple. Of particular note, the result of semen analysis does not represent the final diagnosis but rather the starting point in the diagnostic process of the male partner of an infertile couple. An alteration of semen parameters is a sign of an underlying disorder and different causes, and it might result from different pathophysiological mechanisms [2]. The causes and risk factors of reduced male fertility are numerous and cannot be derived from semen analysis. Therefore, the seminological diagnosis (e.g. reduced sperm count or sperm motility) differs from the clinical diagnosis and a deeper biological understanding of the pathophysiologic process.

As outlined below, semen analysis should always be combined with a comprehensive evaluation to provide definite information on causes and risk factors, genitourinary anatomy, and testicular function. This includes a detailed medical history, physical examination, endocrine assessment, ultrasound examination of the testes and seminal tract, microbiology testing, genetic analysis, and second-level sperm analyses (see below) [2].

It is worth noting that misunderstandings frequently arise among non-experts in reproductive medicine regarding the so-called “reference limits” of semen parameters. Not only should semen analysis be performed by highly experienced personnel in qualified laboratories, but it must also adhere to the World Health Organization (WHO) guidelines [10]. More importantly, the WHO manual does not define what a “normal” semen analysis is, but clearly highlights that the “reference values” reported (in particular the fifth percentile) are not cut-off limits for fertility and infertility. For instance, WHO states that reference values are “not sufficient to establish clinically useful decision limits”, “cannot be used to distinct limits between fertile and subfertile men”, and do not “represent a limit between fertile and infertile men” [10]. Thus, there is a “problem in applying a dichotomous categorization to fertility that must be considered a continuum. It is also well known that there is a substantial overlap of semen examination results between fertile and infertile men” [10]. Finally, WHO recognized that “for an individual patient, a semen analysis is never prognostic of infertility”. In fact, it is well known that natural fertility can occur even with semen parameters below the fifth percentile, while infertility can occur in men with semen parameters above the fifth percentile. Hence, the reference limits should not be used as a specific limit between infertile and fertile men [11]. Importantly, semen examination results below the WHO reference values do not automatically mean that the male partner is the cause of couple’s infertility. Similarly, results above these values do not guarantee that the male partner of an infertile couple is undoubtedly fertile [11]. Additionally, the natural intraindividual variation in semen parameters should always be considered.

In this setting, new technologies or biomarkers could improve the diagnostic work-up of male infertility, involving several emerging techniques. These include genetic testing to identify mutations or chromosomal abnormalities, advanced sperm DNA fragmentation testing, metabolomics for identifying biomarkers in semen plasma, and the use of artificial intelligence to analyse sperm morphology and motility more accurately [12–14]. Additionally, innovations in sperm microfluidics and single-cell RNA sequencing may provide deeper insights into sperm function and fertility potential [15].

In conclusion, semen analysis represents only a surrogate marker of a man’s fertility potential; its results should be clinically interpreted in the context of the other diagnostic procedures in a personalized manner adapted to that particular couple.

## Male partners of infertile couples need a complete andrological workup

As previously mentioned, emerging evidence supports the strong association between male infertility and several risk factors, as well as its possible role as an early indicator for forthcoming cardiovascular (CV) mortality and morbidity [6]. Therefore, a thorough history and andrological and general physical evaluation are mandatory before starting any therapeutical procedure. Semen analysis is a crucial step but cannot replace a comprehensive and specific diagnostic workup.

For example, reduced testes volume along with a reduced, or age-inappropriately increased, prostate volume may raise suspicion of hypogonadism. Testis evaluation can also provide information related to epididymis and the presence of varicocele, which represents a risk factor for sperm impairment.

Ultrasound is a valuable tool that complements physical examination by determining testis volume, structure, and vascularization. Furthermore, given the strong link between male infertility and testis cancer [16] testis ultrasound should be performed in all male patients seeking medical care for couple infertility. In contrast, transrectal and penile Doppler ultrasound should be performed only in specific cases. In particular, transrectal ultrasound is useful to detect distal duct obstruction or abnormalities and provides useful information in subjects with genital tract infection/inflammation. Conversely, penile Doppler ultrasound can allow for a better characterization of subjects with erectile dysfunction [17].

A general physical examination that includes the assessment of body mass index, waist circumference, and blood pressure can rule out possible CV risk factors [2].

A hormonal profile, including the measurement of gonadotropins, total testosterone, and sex hormone-binding globulin plasma levels, can confirm or exclude the presence of clinical (primary and secondary) and subclinical hypogonadism. In addition, elevated follicle-stimulating hormone (FSH) plasma levels, even with normal testosterone levels, suggest a Sertoli cell defect with sperm production impairment (primary testicular damage).

Other hormonal parameters should be assessed only in specific cases. A glycometabolic profile, including serum glucose and lipid levels, is recommended, particularly for patients with overweight or obesity or with other CV risk factors [2, 8, 18].

Standard semen culture should be requested in patients with suspected male genital tract infection/inflammation [2]. Furthermore, moderate evidence suggests performing a Human Papillomavirus DNA test in the semen of infertile patients with asthenozoospermia after excluding other seminal infections [2].

Antisperm antibodies can impair sperm motility and function, contributing to male infertility. They could be evaluated by tests, such as the MAR test and Immunobead Binding Test to identify Aantisperm antibodies on sperm [19].

Genetic analysis, including karyotype and Y chromosome microdeletion, should not be performed routinely, but reserved for specific cases according to current guidelines [2, 8, 18, 20]. Specifically, chromosomal abnormalities and Y chromosome microdeletion can be frequently observed only in men with severe oligozoospermia (< 5 million/mL) compared with the general population. Similarly, CFTR gene mutation analysis should be performed only in men with a documented absence of vas deferens [2, 8, 18]. Other genetic tests, including sperm DNA fragmentation, are not recommended as routine clinical practice but only in specific cases [2, 8, 18].

## Main diagnostic categories and phenotypes of infertile males, and their relation for therapeutic management

In clinical practice, the andrological workup described above is fundamental to identifying the underlying cause of male infertility and deciding whether and how to treat the male partner of the infertile couple. The therapeutic management of an infertile man should aim to remove the etiology of the reduced fertility potential. Box 1 and 2 summarizes the general aims of treatment and the therapeutic strategies in the management of male factor infertility.

**Box 1:** General aims of treatment of male factor infertility

1. ***Restore natural fertility*** The primary goal, when possible, is to restore natural fertility after a comprehensive diagnostic evaluation. This evaluation should include full information on the partner’s fertility potential and risk factors (including age). Treatment should focus on increasing sperm count and quality to facilitate natural conception, regardless of complete “normalization” of semen parameters (i.e., values above the fifth percentile)
2. ***Allow ART and gradual application of ART, improve the outcome of ART*** In cases where natural conception is not feasible for several reasons (for example, female factors and unsuccessful previous treatments), the couple should be addressed to ART. Treatment of the male factor should support the use of ART (e.g., making sperm available in the ejaculate for azoospermic men or retrieving sperm from the epididymis or testes), the gradual application of ART (e.g., intrauterine insemination instead of in vitro fertilization if treatment increases the total number of motile sperm). Furthermore, ameliorating sperm competence can improve the outcome of ART, i.e., a higher pregnancy rate
3. ***Preserve fertility*** Certain causes of infertility, mainly iatrogenic (surgical and medical therapies) and genetic conditions (e.g., Klinefelter syndrome and Y chromosome microdeletions), may require sperm cryopreservation (from semen or testes) to preserve fertility as a precautionary measure. This is particularly critical in cases of azoospermia or when there is a risk of progressive spermatogenic impairment over time. Importantly, fertility preservation is also advisable in idiopathic forms where there is evidence of progressive testicular damage or after therapies that increase the number and quality of ejaculated sperm but pose the risk of regression to pre-treatment situation (e.g., after FSH treatment)
4. ***Cure the underlying disorder and preserve reproductive health*** Numerous medical conditions affect male fertility and reproductive system health, necessitating treatment independent of the couple’s fertility outcome (e.g., diabetes, obesity, lower urinary tract symptoms, benign prostatic hyperplasia, and genital tract infections). Additionally, male infertility could reflect broader health issues of a man and increase the risk of future morbidities (see text). Andrologists are uniquely positioned to assess the general health of male partners in infertile couples, treat underlying disorders, and educate, inform, and play out prevention strategies for future reproductive and overall health. The aim of the management of infertile males should extend beyond having a baby and ensuring ongoing follow-up, particularly in cases of testicular disorders and alterations of the hypothalamic-pituitary-testis axis

**Box 2:** General therapeutic strategies of male factor infertility

1. ***Etiologic management (remove the cause)*** When possible, remove the cause of infertility. This means that the diagnostic process has clearly identified the underlying etiology. Examples include treatment of a prolactinoma or hypogonadotropic hypogonadism, semen infections, or erectile dysfunction. Evidence supporting the efficacy of this strategy is very high
2. ***Oriented management (act on risk factor)*** If a clear causal factor cannot be identified, but risk factors for reduced fertility are present, an oriented management approach can be applied to mitigate them. Examples include weight management in cases of overweight/obesity, smoking cessation, or discontinuation of anabolic steroid use. Evidence supporting the efficacy of this strategy is modest
3. ***Goal-oriented management (bypass the problem)*** In cases where no clear causes or significant risk factors are identified (e.g., idiopathic forms) or when the cause cannot be removed, the problem can be bypassed through techniques such as sperm retrieval for ART. Examples include testicular sperm extraction for primary hypergonadotropic testicular damage (e.g., Klinefelter syndrome) or sperm retrieval from urine in cases of retrograde ejaculation
4. ***Empirical management*** This approach aims to increase the quantity and quality of sperm when etiologic and oriented managements are ineffective or inapplicable, particularly in idiopathic forms. Empirical treatment may target testicular function (to improve spermatogenesis), seminal tract, or sperm function. Examples include treatment with FSH (strong evidence of efficacy), anti-inflammatory, and antioxidants/nutraceuticals (low evidence of efficacy)

Many classification attempts of male infertility have been made based on various factors such as underlying causes, types of abnormalities, and specific conditions affecting fertility. From a clinical perspective, the most commonly used classification consists of three categories of male infertility based on detectable causes of sperm production impairment [21]. The first group consists of pre-testicular forms, which include conditions where the testes are not adequately stimulated by the hypothalamic-pituitary axis. In these cases, the spermatogenetic potential of the testes is normal, but hormonal stimulation on the gonads is insufficient, as seen in hypogonadotropic hypogonadism. The second group encompasses testicular causes of male infertility, where the testes cannot produce normal amounts of sperm and hormones due to intrinsic dysfunction, such as genetic abnormalities, infections and inflammation, torsion, trauma or injury, and cancer. The third group comprises post-testicular conditions, where the hypothalamic-pituitary–gonadal axis functions properly, but sperm cannot reach the oocyte. This is typically due to obstructions in the reproductive tract (both congenital and acquired) and ejaculatory or erectile dysfunctions, which impede the sperm's exit.

While this classification serves research purposes by standardizing the diagnosis of male infertility, it has limited utility in clinical settings and does not lead to uniformly allocated treatment options. Each category encompasses different causes and risk factors, making a standardized therapeutic approach impractical. For instance, male infertility due to testicular infection or varicocele are both classified as “testicular causes”; however, their available and effective treatments are extremely different, ranging from antibiotic therapies [22, 23] to surgical varicocele resolution [24–27].

Given this limitation, there is a need for a new classification system that can better guide treatment options. Here, we propose new diagnostic categories that are more useful for classifying male infertility based on pathophysiologic mechanisms and guiding treatment choices: (i) Inflammation and infection of the genital tract; (ii) duct obstruction (both congenital and acquired, including retrograde ejaculation); (iii) primary testicular damage of different degrees, which is further divided into hypergonadotropic (elevated FSH plasma levels) and normogonadotropic (normal FSH plasma levels); (iv) secondary testicular dysfunction (i.e., hypogonadotropic hypogonadism); (v) idiopathic semen alteration (where potential causes are no detectable); (vi) unexplained infertility (where no semen analysis alterations are found, despite the couple infertility). This six-group etiological classification unifies the causes of male infertility according to the appropriate therapeutic approach. Men in the first group should be treated to reduce inflammation and resolve any present infections. In the second group, male infertility can be addressed surgically by resolving or bypassing obstructions to facilitate sperm exit; alternatively, sperm can be retrieved from the epididymis, testes, or urine in the cases of retrograde ejaculation. The third group is probably the most challenging, as treatments to restore spermatogenesis are unavailable in the hypergonadotropic subgroup; however, the normogonadotropic subgroup could be empirically treated, which will be discussed further below. The fourth group focuses on restoring pituitary stimulation of the testes. The fifth group could be treated with empirical therapy, which will also be discussed further. The sixth group represents significant challenges in human reproductive medicine, as no clearly effective therapeutic approaches have been identified so far.

To note, this classification is intended primarily for therapeutic management and considers simple aspects (patient history, clinical signs of hypogonadism, testis volume, semen alterations, FSH, and testosterone levels). Comprehensive diagnostic management is still necessary to provide an etiologic and pathophysiologic explanation of infertility, as outlined above.

## The problem of the definition of “idiopathic” infertility

Male factors account for 50% of all cases of a couple’s infertility. Several causes have been ascribed in the context of male factor infertility, ranging from clinical, hormonal, and genetic conditions. However, almost 30% of men still exhibit impaired sperm parameters without any identifiable cause, a condition referred to as idiopathic male infertility [8, 28, 29]. The individual sperm parameter provides only a partial indication of actual fertility potential. In this context, data would suggest that normal sperm parameters do not reliably predict fertility in a real‐life setting [30–32]. Between 15 and 40% of men are infertile despite having normal sperm parameters, normal medical history, and normal physical examination; this condition is currently defined as unexplained male infertility [33]. Therefore, the diagnosis of idiopathic infertility is a diagnosis of exclusion in male factor infertility cases.

The definition and prevalence of idiopathic infertility vary consistently across previously published reports, depending on the possible postulated causal factors and the baseline diagnostic workup selected by the investigators [34]. Of relevant note, a definition of idiopathic infertility is often overused in everyday clinical settings. In reality, this term should be reserved for cases where no cause can be identified after a detailed and meticulous diagnostic process. Both idiopathic and unexplained male factor infertility types are exclusionary diagnoses, meaning that an extensive andrological approach is necessary to exclude causes, risk factors, problems in the testicular function, and genitourinary anatomy. Despite the unclear underlying causes and biopathology of both idiopathic and unexplained infertility, there has been no in-depth investigation into the specific clinical and laboratory characteristics of infertile men belonging to these two groups. Indeed, it has previously been shown that a more accurate and comprehensive workup can improve the diagnostic process by increasing its accuracy during the clinical evaluation of the infertile male. Ventimiglia et al. performed an extensive diagnostic workup in 1,147 white-European men with male factor infertility belonging only to couples complaining of primary infertility to properly and precisely assess the possible underlying causal factors. By employing a comprehensive workup, they found a causal category for 81% of the study cohort. Notably, men with less severe male factor infertility were the most likely to lack a clear causal identification [32].

Recent evidence suggests that male idiopathic infertility may be linked to several unidentified pathological conditions that may perturb the testicular micro-environment and spermatozoa characteristics (e.g., pollution exposure, reactive oxygen species), leading to DNA damage and genetic/epigenetic abnormalities. These factors ultimately decrease overall sperm quality and fertility potential. In light of this, leading international scientific societies strongly recommend that every infertile man should undergo a detailed medical history review, an accurate physical examination, and at least one semen analysis adhering to WHO reference values to screen for potential causes of male infertility. As outlined above, routine semen analysis is one of the cornerstones of male factor infertility investigation, being significantly related to conception chances [7, 8]. However, the individual semen parameter provides only partial insight into actual fertility potential and cannot be used alone to define unexplained infertility [35, 36].

A major issue with idiopathic male factor infertility is that it does not support a specific treatment workup in everyday clinical practice. Andrologists working in reproductive medicine often encounter a lack of effective therapeutic options for male factor infertility patients and a misclassification of the disease etiology. This has several drawbacks regarding further diagnostic workup and treatment [37, 38]. Additionally, identifying possible causal factors could offer psychological relief to the infertile couple.

Despite several advancements in the last few years, assessing and treating infertile couples remains unsatisfactory in a relevant proportion of cases [1]. The need for additional tests in any case of idiopathic male factor infertility has been increasingly recognized. Recently, the Guidelines Group on Unexplained Infertility from the European Society of Human Reproduction and Embryology (ESHRE) [36, 39] recommended intrauterine insemination combined with ovarian stimulation as the first-line treatment for couples with idiopathic infertility, thus effectively precluding any real attempt to investigate the male’s condition within the couple.

## Male infertility as a mirror of general health and predictive marker of future health

As widely discussed, infertility is a disease of near-endemic proportions, affecting up to 15% of couples of reproductive age. Growing epidemiologic and clinical data indicate that male factor infertility is not merely an epiphenomenon of other diseases but a disease per se, with relevant clinical implications and health-related outcomes [7, 8]. Indeed, it has become evident that infertile men tend to be less healthy than fertile men of similar age and ethnicity.

As for comorbidities, such as non-communicable diseases (NCDs), associated with infertility, a classification has been proposed. Comorbidities are considered prevalent if the disease is present before or within 12 months from the infertility diagnosis and incident if the comorbidity is diagnosed ≥ 12 months after the infertility diagnosis [40].

Associations have been described between infertility and prevalent non-malignant chronic diseases [32, 38, 41, 42], such diabetes, metabolic syndrome, obesity, and CV diseases [4, 5]. Moreover, growing evidence supports the link between male infertility and the risk of prevalent malignant diseases [16, 43, 44]. These findings align with large population-based studies suggesting a higher overall mortality risk for infertile men [45]. Quantitative measures of the overall health status have also been correlated with alterations, mostly quantitative, of sperm parameters [46]. Similarly, recent data suggest that infertile men face an increased risk of developing unfavorable age-related incident comorbidities in almost 10% of cases [6, 47, 48].

Despite the emerging and robust epidemiological evidence, the pathogenetic link between infertility and overall male health status remains elusive, and the mechanisms fostering the two require further elucidation. Although the endocrine function of the testis (i.e. hypogonadism) might represents a possible explanation, comorbidities are frequent also in infertile men with normal testosterone concentrations [4]. Preliminary evidence indicates chronic inflammation and early senescence among the possible biopathological links between male infertility and comorbid condition [49, 50].

While the primary goal of fertility assessment in the male is to optimise reproductive potential and achieve paternity, being infertile does not only pose a clinical problem in terms of reproductive outcomes. This highlights the need for an extensive diagnostic work-up [51] to identify potential causes and possible effective treatments to increase the chance of live births. Moreover, fertility assessment is an opportunity to screen those men who may harbour occult disease, providing an opportunity to mitigate the long-term risk of incident NCDs. Identifying specific biopathologic signatures underlying both male factor infertility and overall men’s health could have relevant real-life consequences for promoting personalized preventive strategies. Such an approach could improve the physical and psychological health of infertile men while also offering cost-effective impact on global health systems.

Thus, andrologists with relevant expertise in the field of reproductive medicine for men with male factor infertility have the responsibility not only to pursue effective and innovative therapeutic options for infertility but also to implement ad-hoc follow-up and screening programs promoting better health strategies.

## Evidence-based treatments: guidelines on FSH therapy

Currently, several guidelines are available in the literature to assist clinicians in the decision-making process for male infertility management. Some guidelines consider male infertility in its complexity [8, 52, 53], while others specifically focus on specific subgroups, such as men with semen alterations, idiopathic infertility [18, 54] or azoospermia [55]. Although these guidelines emphasize the importance of evaluating the male partner of infertile couples, they do not agree on many aspects of the available therapeutic options. In particular, the pharmacological treatments described in these guidelines are not aligned and seems extremely challenging. The condition described as idiopathic infertility, where an etiological treatment is not available since the cause of the condition is not detectable, presents the most relevant clinical challenge. Indeed, in this condition, an empirical treatment aimed at stimulating spermatogenesis is suggested for these patients. However, the strength of this recommendation varies among guidelines, and the level of evidence reported is generally low.

In particular, the European Association of Andrology (EAA) and in the Italian Society of Andrology and Sexual Medicine (SIAMS) guidelines [18, 54], suggest the FSH administration to men with idiopathic infertility. Although the level of evidence is low, these guidelines suggest FSH treatment in selected men from infertile couples affected by normogonadotropic (normal FSH plasma levels) oligoasthenoteratozoospermia (OAT), to improve quantitative and qualitative sperm parameters and pregnancy rate, whether natural or through ART. Similarly, the European Association of Urology (EAU) guidelines suggest FSH treatment in men with idiopathic oligozoospermia and normal FSH plasma levels [8]. On the contrary, the American Urological Association (AUA)/American Society for Reproductive Medicine (ASRM) guidelines only suggest FSH treatment in idiopathic infertility [56]. Importantly, only the Italian guidelines [2] apply more stringent selection criteria to maximize treatment effectiveness, specifically men with oligozoospermia and/or asthenozoospermia (not only idiopathic), FSH plasma levels below 8 IU/L and no sign of obstruction of the seminal tract. This statement is very important, as the two conditions (alterations in semen parameters due to reduced spermatogenic activity and those due to sub-obstruction of the seminal tract) are indistinguishable unless detailed analyses are performed. The determination of the appropriate cut-off for high FSH serum levels remains challenging [57], and the suggested threshold of the Italian guidelines are mainly based on normative aspects rather than evidence from clinical studies. Nevertheless, it could represent a valid threshold above which FSH administration should be avoided because of likely ineffectiveness.

In clinical practice, only in Italy FSH is available (and its cost is covered by the National Health System) for men with infertility, otherwise hypogonadotropic hypogonadism. Despite this, a recent Italian survey demonstrated that only 55% of 718 patients eligible for FSH treatment under national rules were effectively treated [58].

Of note, the empirical administration of anti-oxidants for male infertility is neither recommended nor suggested by clinical guidelines due to the low level of evidence [2, 8, 18, 56]. However, these treatments are over-utilized and prescribed world-wide [59]. A recent Cochrane meta-analysis suggested that anti-oxidant supplementation in male infertility could improve live birth rates for couples attending assisted reproduction [60]. This result, however, must be carefully considered, since it was obtained by only 12 studies and did not consider any confounding factors [54]. Accordingly, the use of anti-oxidants could be suggested in selected patients with idiopathic semen analysis alterations and/or high oxidative stress, since in some cases, they might improve sperm parameters [54].

## Treatment with FSH: current status

The use of FSH for treating idiopathic male infertility stems from its success in managing hypogonadotropic hypogonadism. In this condition, FSH administration, combined with hCG, is known to significantly restore spermatogenesis and improve pregnancy chances [61, 62]. This therapeutic approach has been extended to male infertility due to the physiological role of FSH in Sertoli cell function and spermatogenesis [63]. The main action of FSH is on the first, mitotic, phases of spermatogenesis [64], with the expected effect being a quantitative increase of spermatogenesis and semen parameters. However, it is crucial to distinguish between these two conditions and their treatment strategies. In hypogonadotropic hypogonadism, FSH administration acts as replacement therapy, compensating for the lack of physiological pituitary stimulation. In contrast, for infertility, the pituitary stimulation is not deficient, and thus FSH administration should serve as an overstimulation rather than a replacement. This approach is intended to stimulate the testicular reserve function. Several research indicated that even in severe cases of azoospermia [65], such as in Klinefelter syndrome [66–68], histological evaluations can reveal residual spermatogonia, suggesting the presence of a potential spermatogenesis reserve. Future research should focus on identifying predictive markers of testicular function and dysfunction to better select candidates for FSH treatment. Similar to the female counterpart, there is the urgent need in male fertility to identify a potential testicular reserve function marker.

FSH administration for men with idiopathic infertility has been evaluated in over 20 clinical trials, summarized in four different meta-analyses [69–72]. These studies collectively suggest that FSH administration significantly increases sperm production (in terms of sperm concentration and total sperm count) [73] in a dose-dependent manner [74]. Moreover, this treatment has been shown to significantly increase pregnancy rates, both spontaneously and through ART. However, while FSH treatment is effective, the available data suggest that it must be refined and reconsidered. The number-needed-to-treat (NNT) to achieve one pregnancy ranges from 10 to 18 men.

These finding implies that FSH can be effective for treating male infertility but should be approached with a different rationale (shifting from replacement to stimulation) and following careful patient selection to maximize its efficacy. Patient selection should consider baseline FSH levels, absence of seminal tract obstruction, and possibly other parameters to distinguish different subgroups of infertile patients who might respond differently to treatment [75–78].

Of note, no side effects or adverse events were ever reported as a consequence of FSH treatment in males [78], differently from women in which and excess of FSH might result in ovarian hyperstimulation syndrome (OHSS). Furthermore, FSH-secreting tumors in females lead to OHSS, whereas in males no other effects than increasing testis size is observed. Possible extragonadal effects of FSH are a very much debated issue and, for the time being, should not be a concern [78].

## Focus on treatment with FSH: drawbacks

Despite the data supporting the role of FSH treatment in subjects with idiopathic infertility, clinical practice has shown variability in patient response, with some individuals experiencing improvements in semen parameters (responders) and others showing no change (non-responders). Unfortunately, reliable clinical or biochemical markers are currently lacking to differentiate between these two populations before starting treatment. As a result, the efficacy of FSH treatment could be assessed only after about 3 months through semen analysis, as no earlier biochemical/molecular markers of efficacy are available.

To better understand this issue and improve FSH treatment outcomes in male patients with couple infertility, it is important to analyze some molecular aspects of FSH. The latter is a complex heterogeneous glycoprotein resulting from α and β subunit glycosylation at pituitary level [79]. Available data have clearly documented that the degree of FSH glycosylation can influence its receptor binding activity, being the more glycosylated isoforms less effective in term of receptor biding and signal transduction [79]. However, no specific outcome data for testing treatment using different FSH glycoforms are available.

In addition, data from women undergoing ART have revealed the possible presence of several genetic polymorphisms in either the FSH gene and its receptor, which can modulate FSH treatment outcomes [79]. For examples, lower testis volume and sperm quality was reported in subjects with FSH receptor pN680S homozygous S [80, 81]. Similar data have also been documented for FSH gene polymorphism [80, 82]. However, only a study have tested FSH outcomes according to FSH or FSH receptor genetic profile [79].

Besides genetic considerations, it is possible that some patients may need higher FSH dosage and/or longer therapy duration, while others may also respond to a lower FSH dosage.

Unlike women, where a higher dosage of FSH can result in ovarian hyperstimulation syndrome, no dose-dependent side effects have ever been reported in men [79]. In line with this aspect, limited evidence from a single group provided better outcomes with higher FSH dosages (200 IU or 300 IU of rhFSH) on alternate days for 3 months [82]. However, it is important to note that the aforementioned available study is not sufficiently powered to draw final conclusions.

Other open questions include the analysis of proposed baseline FSH thresholds or the concomitant use of hCG. Italian guidelines recommend limiting the use of FSH only in those subjects with baseline FSH plasma levels below of 8 IU/L. It is worth noting that this threshold is more empirical than evidence-based, and no definitive cut-off can be suggested at present. However, a recent meta-analysis showed that elevated gonadotropin levels blunted the efficacy of hormonal therapy prior to surgical sperm retrieval in non-obstructive azoospermia, confirming the limited role in hypergonadotropic patients [83].

In conclusion, the major challenge related to FSH treatment is the variable (and unpredictable) response. In our opinion, this is due to different factors: suboptimal phenotype characterization, incomplete identification of responders before treatment, absence of biomarkers of response during treatment, and treatment regimens (dose, interval, duration) based on evidence from hypogonadotropic hypogonadism, which indeed represents a distinct condition from infertility (Box 3).

**Box 3:** Treatment with FSH: perspectives

– ***Selection of candidates to therapy*** Potential responders to FSH treatment can be selected based on a few clinical and diagnostic criteria: semen analysis (including DNA fragmentation when indicated), endocrine evaluation (FSH and testosterone plasma levels), and comprehensive evaluation to rule out obstructive/sub-obstructive forms (testicular volume, scrotal and transrectal ultrasound). Importantly, the decision to administer FSH treatment to the male partner of infertile couples should also consider the fertility status of the female partner. The variable response rates, even among carefully selected patients, highlight the need for better clinical characterization and validated biomarkers in the future. The improvements could help identify the right candidates, increase the response rate, and allow individualized doses and treatment duration adjustments. The recent development of the so-called Aphrodite criteria represents the first effort in this direction by identifying different classes of infertile men who may benefit from FSH ± hCG therapy
– ***Factors that might predict the response*** Several efforts have been made to identify factors to distinguish, before starting the treatment, between patients who might or might not respond to FSH therapy [78]. Seminal parameters (e.g., high *vs* low spermatid count), testicular cytology (e.g., hypospermatogenesis *vs* spermatogenic arrest), and FSHB/FSHR gene polymorphisms are promising areas of research; however, the evidence to date preclude their routinely clinical applications. In the future, miRNA or protein markers in semen or sperm might be used to create a tailored FSH therapy plan [78]. Personalizing FSH treatment is mandatory to minimize side effects, avoid wasted time with ineffective therapies, and improve efficacy by predicting the optimal dose and duration of treatment
– ***Markers of FSH efficacy*** Clinical and biochemical/molecular markers of response to FSH treatment need to be identified, as to date, the response can only be verified with semen analysis after 3 months of therapy. The availability of early biomarkers of FSH action would allow real-time adjustments in therapy, similar to how the treatment is personalized during ovarian hyperstimulation for multifollicular growth in ART. This would enable more precise dosing and treatment duration
– ***From substitutive treatment to pharmacologic hyperstimulation*** As discussed in the main text, the rationale and treatment protocols for FSH in male infertility are derived from evidence in men with hypogonadotropic hypogonadism, where FSH plays a physiological role in stimulating quantitatively and qualitatively normal spermatogenesis. However, these two conditions differ significantly. Infertile men are not hypogonadal but rather suffer from quantitative and/or qualitative problems in spermatogenesis that may benefit from additional stimulus. Therefore, while treatment for hypogonadotropic hypogonadism is a typical replacement therapy, treatment for male infertility should be regarded as a hyperstimulation. This rationale is supported by evidence that spermatogenesis does not always function at full capacity in physiological conditions, and pharmacological doses of FSH might boost the “testicular reserve”. Numerous studies in animals and humans support this concept: after hemicastration, the remaining testis compensates with increased volume [84–89], as seen after unilateral orchiectomy for testicular cancer [90]; men with FSH-secretig pituitary adenoma have increased testis volume [91, 92]; FSH treatment increases testicular volume in men [93]; FSH treatment increases spermatogenesis in the adult monkey [94] and increases spermatogonia number in men [64]
– ***New clinical scenario*** As described in the main text, FSH treatment in infertile men has an evidence-based efficacy in terms of enhancing semen parameters and pregnancy rate, although there is still room for better characterization to increase response rates. However, it is still under-utilized, both with the aim of restoring natural fertility, and more importantly for increasing the outcome of ART. To this regard, short-term (one month) FSH treatment to reduce sperm DNA fragmentation is an important aspect. Another area to explore in future studies is the association of FSH with other therapeutic strategies, both in idiopathic patients (e.g. combination with hCG or antioxidants) and in patients with co-morbidities (e.g. weight management or antidiabetic drugs)

## Conclusion

This review and expert opinion aimed to highlight the current drawbacks and future perspectives in diagnosing and treating male factor infertility, focusing on the only evidence-based medical therapy currently available, namely FSH supplementation. Box 4 summarizes the main statements regarding diagnosis, general therapeutic strategies and treatment with FSH elaborated for this panel of experts.

**Box 4:** Statements of experts on male factor infertility

***Diagnosis***
− Infertility is not a matter of the woman or of the man, it’s a problem involving a couple. Andrologists and gynaecologists should proceed jointly in the management, a typical malpractice being focusing the attention just to one of the partners
− The fertility potential of each partner and/or specific conditions affecting a member of the couple influence the clinical and treatment approach
− The clinical approach to male factor infertility should go far beyond semen analysis. Male infertility might be due to a variety of conditions and risk factors, and the seminological diagnosis should not be confused with a diagnosis based on etiological identification and pathophysiological mechanisms
− The result of semen analysis should not be interpreted as absolute markers of (in)fertility and the so-called reference limits are a general indication of the fertility potential
− Idiopathic infertility is an exclusion diagnosis. It can be applied only after a detailed diagnostic process
− Male infertility might reflect the general health of a man and increases the risk of future morbidities. Therefore, the identification of subjects with signs of spermatogenic and endocrine testicular impairment is important for their follow up and prognosis, independently from their ability to conceive naturally or by ART
***General therapeutic strategy***:
− Only an extensive diagnostic process could direct the treatment strategy and influence prognosis
− For therapeutic management the main factors to consider are history, clinical signs of hypogonadism, testis volume, semen alterations, FSH and testosterone levels, but definition of the causes and risk factors present are important in defining the best treatment strategy
− Main diagnostic categories useful for the choice of treatment are: (i) Inflammation and infection of the genital tract; (ii) Duct obstruction and retrograde ejaculation); (iii) Primary testicular damage (hypergonadotropic and normogonadotropic); (iv) Secondary testicular dysfunction (hypogonadotropic hypogonadism); (v) Idiopathic semen alteration; (vi) Unexplained infertility
− The general aims of treatment are: (i) if possible, restore natural fertility; (ii) allow ART, allow gradual application of ART, improve ART success; (iii) preserve fertility; (iv) cure the underlying disorder and preserve reproductive health
− The therapeutic strategies for male factor infertility are: (i) Etiologic management (remove the cause); (ii) Oriented management (act on risk factor); (iii) Goal-oriented management (bypass the problem); (iv) Empirical management (by acting on testicular function, seminal tract or sperm function
***Treatment with FSH***
− FSH is the best evidence-based treatment option for empirical management of male factor infertility, but it is under-utilized
− To date, treatment with FSH is suggested for men with oligozoospermia and/or asthenozoospermia with normal FSH plasma levels and no sign of obstruction of the seminal tract
− Treatment with FSH in selected patients improves semen parameters and pregnancy rate, both naturally and with ART. However, the response is variable, due to the absence of robust clinical and biochemical/molecular markers predictive of efficacy
− Treatment with FSH should be considered as a hyper-stimulation strategy rather than a replacement therapy approach, which is used for hypogonadotropic hypogonadism
− Treatment with FSH should consider both long-term approach (at least 3–4 months) to booster spermatogenesis exploiting the “testicular reserve”, and short-term treatment (1 month) to increase sperm quality for ART

**Fig. 1:**
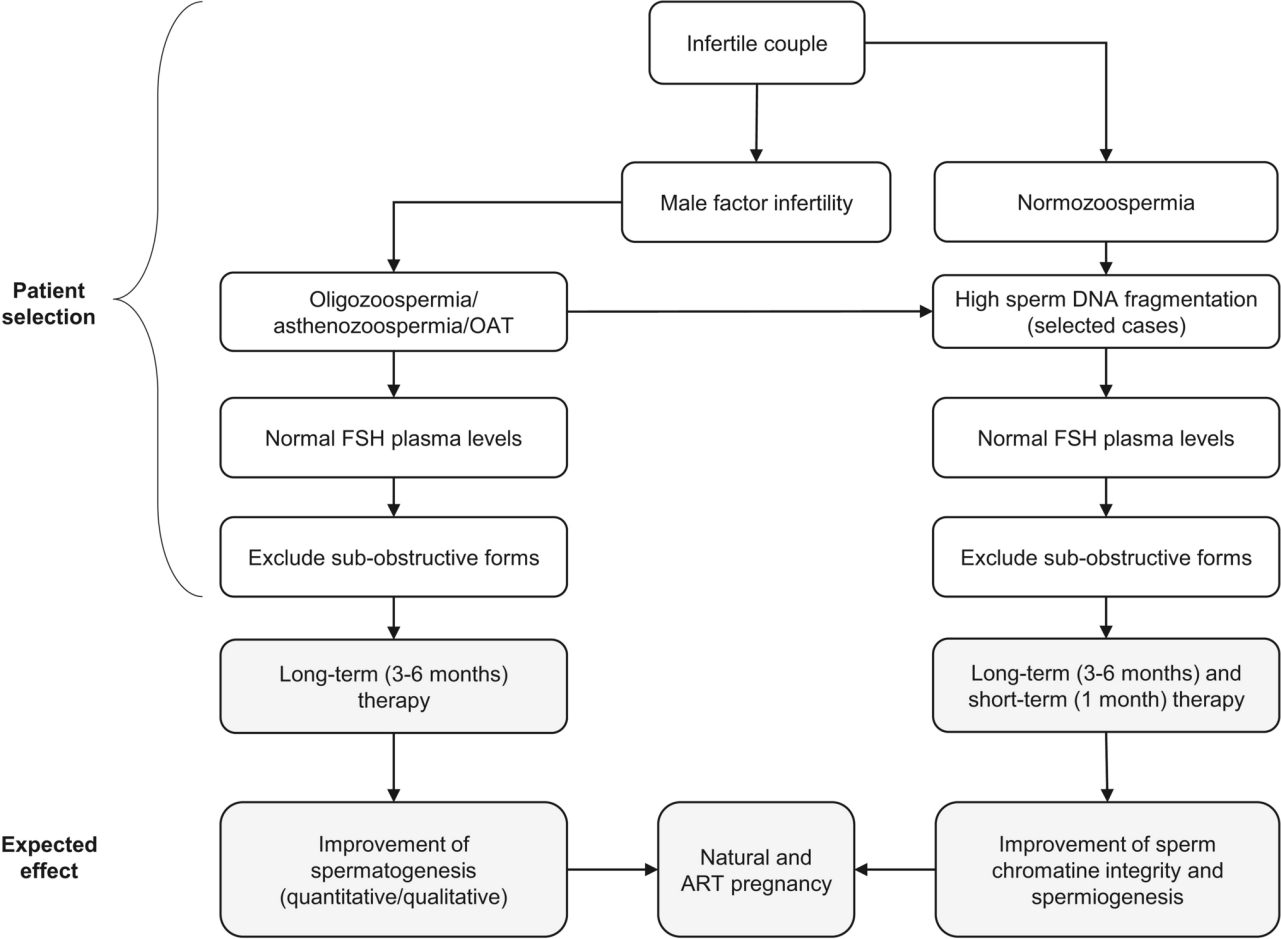
Selection of candidates to FSH therapy and expected outcomes

A thoughtful, comprehensive management approach is the prerequisite for an etiological and pathophysiological diagnosis that can indicate the best treatment strategy, which should be individualized to the patient and couple characteristics. We propose a new practical classification of male factor infertility that could direct treatment and suggest future directions for better management of patients eligible for FSH therapy (Fig. 1). Table 1 represents a schematic flowchart of management of male factor infertility, including the new classification of male infertility in diagnostic categories useful in defining the best therapeutic strategy.

**Table 1:** Schematic flowchart of management of male factor infertility

Step	
1. Assess the causes and risk factors	• History and physical examination • Fertility potential of the female partner
2. Assess the fertility potential	• Semen analysis
3. Proceed to full investigation to define etiology, pathophysiology and to have prognostic and therapeutic information	Consider: • Infections • Endocrine assessment • Scrotal and trans-rectal ultrasound • Testicualr cytology/histology • Genetic testing
4. Classify in diagnostic categories to define the best therapeutic strategy	• Inflammation and infection of the genital tract • Duct obstruction and retrograde ejaculation • Primary testicular damage (hypergonadotropic or normogonadotropic) • Secondary testicular dysfunction (hypogonadotropic hypogonadism) • Idiopathic semen alteration • Unexplained infertility
5. Select the treatment strategy	• Etiologic management (remove the cause) • Oriented management (act on risk factor) • Goal-oriented management (bypass the problem) • Empirical management (by acting on testicular function, seminal tract or sperm function)
6. Define the aims of treatment	• If possible, restore natural fertility • Allow ART • Allow gradual application of ART • Improve ART success • Preserve fertility • Cure the underlying disorder and preserve reproductive health

Given the high prevalence of couple infertility, the number of conditions and risk factors for reduced male fertility and testicular function, and the pivotal paradigm that infertility reflects a man’s overall health and increases the risk for future morbidities, we believe greater efforts are needed in this field. In the near future, we hope there will be more awareness among general practitioners and specialists, establishing reference centres and clinical networks based on the hub-and-spoke model, and more basic and clinical research, including multicentre clinical trials.

## Data Availability

Data sharing not applicable to this article as no datasets were generated or analysed during the current study.
